# A role for sirtuin 1 in FGF23 activation following β-glycerophosphate treatment

**DOI:** 10.1007/s00424-024-02974-5

**Published:** 2024-05-21

**Authors:** Danielle M. A. Ratsma, Max Muller, Marijke Koedam, M. Carola Zillikens, Bram C. J. van der Eerden

**Affiliations:** Laboratory for Calcium and Bone Metabolism and Erasmus MC Bone Centre, Department of Internal Medicine, Erasmus MC, Erasmus University Medical Center, Room Ee585b, Dr. Molewaterplein 40, 3015 GD Rotterdam, The Netherlands

**Keywords:** Fibroblast growth factor 23, Osteocytes, Phosphate, Reactive oxygen species, Nutrition, Sirtuin 1

## Abstract

Phosphate homeostasis is vital for many biological processes and disruptions in circulating levels can be detrimental. While the mechanisms behind FGF23 regulation have been regularly studied, the role of extracellular phosphate sensing and its impact on fibroblast growth factor 23 (FGF23) expression remains unclear. This study aimed to investigate the involvement of reactive oxygen species (ROS), silent information regulator 1 (SIRT1), and Hairy and Enhancer of Split-1 (HES1) in regulating FGF23 in FGF23 expressing MC3T3-E1 cells. MC3T3-E1 cells treated with β-glycerophosphate (BGP) resulted in increased *Fgf23* expression. Inhibition of ROS formation by inhibition of NADPH oxidase, which is essential for ROS production, did not affect this response to BGP, suggesting ROS is not involved in this process. Moreover, treatment with tert-butyl hydroperoxide (TBHP), a ROS-inducing agent, did not increase *Fgf23* expression. This suggests that ROS machinery is not involved in FGF23 stimulation as previously suggested. Nonetheless, inhibition of SIRT1 using Ex527 eliminated the *Fgf23* response to BGP, indicating its involvement in FGF23 regulation after BGP treatment. Indeed, activation of SIRT1 using SRT1720 increased *Fgf23* expression. Moreover, transcription factor *Hes1* was upregulated by BGP treatment, which was diminished when cells were treated with Ex527 implying it is also regulated through SIRT1. These findings suggest the existence of an upstream SIRT1-HES1 axis in the regulation of FGF23 by phosphate, though we were unable to find a role for ROS in this process. Further research should provide insights into phosphate homeostasis and potential therapeutic targets for phosphate-related disorders.

**Supplementary Information:**

The online version contains supplementary material available at 10.1007/s00424-024-02974-5.

## Introduction

Phosphate is an essential nutrient as it is indispensable for most biological processes [18]. Since excess or deficiency of phosphate can have a negative impact on these processes, organisms have developed systems to maintain phosphate homeostasis. In mammals, great progress has been made in understanding phosphate homeostasis but the mechanism of sensing changes in extracellular phosphate levels is still poorly understood [1, 8, 18].

Serum phosphate levels are tightly regulated by a bone-kidney-gut axis involving the proteins fibroblast growth factor 23 (FGF23) and α-klotho [22]. FGF23 is secreted by osteocytes in the bone and binds to its receptor fibroblast growth receptor 1 (FGFR1) and co-receptor α-klotho in the kidney where it inhibits the reabsorption of phosphate and the conversion of 25-hydroxy vitamin D (25(OH)D) to 1,25-dihydroxy vitamin D (1,25(OH)_2_D) and increases the degradation of 1,25(OH)_2_D [13]. Moreover, FGF23 inhibits the secretion of parathyroid hormone (PTH), while PTH is able to stimulate the expression of FGF23 and the conversion of 25(OH)D to 1,25(OH)_2_D [3] further emphasizing the accurate control of phosphate homeostasis. Klotho deficient mice (klotho(-/-)) have a short lifespan, which can be rescued when the renal type-2 sodium phosphate transporters are knocked out (NaPi2a(-/-)/klotho(-/-) mice), indicating that defects in the FGF23-klotho axis leading to premature ageing occur through increased serum phosphate levels [16, 20]. This is supported by the findings that a premature ageing phenotype in Fgf23^−/−^ mice is rescued by a low phosphate diet [19, 28].

In the past, reactive oxygen species (ROS) were predominantly described as accelerators of ageing, but more recent studies have shown that they are important cellular signaling molecules. More specifically, low levels of ROS are essential for different biological processes, while high levels of ROS will results in cellular damage or death [12]. Studies in endothelial cells demonstrated that elevation of extracellular phosphate levels leads to increased ROS production, which was inhibited upon blockage of the sodium-dependent phosphate transporters (PiTs) [26]. Studies in the osteosarcoma cell line UMR106 showed that phosphate stimulated *Fgf23* expression via ROS production. When ROS production was inhibited using apocynin, *Fgf23* failed to be increased by phosphate treatment. Moreover, the authors showed that cells treated with H_2_O_2_ also increased *Fgf23* expression in the absence of phosphate indicating the necessity of ROS production for FGF23 function [14]. A study in mice revealed that exercise induces *Fgf23* expression and secretion and that FGF23 protected against exercise-induced ROS production as treatment with FGF23 decreased ROS production and increased endurance. Taken together, these results indicate that there is a functional feedback mechanism between FGF23 and ROS [17].

Silent information regulator 1 (SIRT1) is a ubiquitously expressed protein and has a role in the prevention of ROS. Moreover, SIRT1 is a conserved nutrient sensor and longevity associated protein [23]. Many studies have established that SIRT1 is a potent intracellular inhibitor of oxidative stress by regulation of the expression of anti-oxidant genes [27]. During oxidative stress SIRT1 deacetylates forkhead box O3 (FOXO3a) resulting in the transcription of FOXO3 target genes [6, 25]. SIRT1 has also been described as a metabolism sensor in varying tissues [7]. It acts as a glucose sensor in neural stem and progenitor cells through regulation of the transcription factor hairy and enhancer of split-1 (*Hes1*) [11]. In a high glucose environment SIRT1 deacetylates and represses transcription of HES1 while in a low glucose environment *Hes1* transcription is activated, leading to cellular self-renewal [11]. Interestingly, the osteocyte transcriptome is very similar to the neuronal transcriptome and knowledge of neuronal networks may improve the understanding of the osteocyte network [32]. *Hes1* has also been described as part of the osteocyte transcriptome, but it is unknown whether it has a function in response to glucose concentrations [32]. Since previous studies observed a correlation between the upregulation of *HES1* and *FGF23* [29], and ROS has a known stimulatory effect on FGF23 and HES1 [14, 30], we hypothesized that a ROS-SIRT1-HES1 axis may be involved in phosphate sensing and subsequent regulation of the FGF23 response.

Using FGF23 expressing MC3T3-E1 cells we aimed to gain deeper understanding in the function of ROS in FGF23 regulation following phosphate treatment and the potential role of SIRT1 in this process.

## Materials and methods

### Cell culture

The murine pre-osteoblastic MC3T3-E1 cell line was cultured and passaged in proliferation medium, namely alpha-minimum essential medium (αMEM; A10490-01, Gibco, Paisley, UK), supplemented with 10% fetal bovine serum (Gibco), 100 Units/mL penicillin and 100 µg/mL streptomycin (Gibco). Cells were used until passage 23. For experiments, 2.0*10^4^ cells were seeded in 12-wells plates and kept for 2 days in proliferation medium before being switched to osteogenic medium (αMEM, 10% FBS, 100 Units/mL penicillin and 100 µg/mL streptomycin, 10 mM β-glycerophosphate (BGP, Sigma-Aldrich, Missouri, United States), 50 µg/ml ascorbic acid (Sigma-Aldrich) and 0.1 µM dexamethasone (Sigma-Aldrich) as described previously [21, 31]. Cells were differentiated into osteocyte-like cells for 21 days, after which β-glycerophosphate (BGP) was removed from the differentiation medium and cultures were continued until day 28. Cells were treated for 24 h, unless indicated otherwise, before being lysed to obtain total RNA or cell lysates as described below. Cells were treated with 4 mM BGP, 500 µM apocynin, 50 µM Ex527, 1 µM SRT1720, 4 mM Sodium phosphonoformate tribasic hexahydrate (PFA), 5, 10 or 50 µM tert-butyl hydroperoxide (TBHP), 10 mM D-glucose or a combination (all from Sigma Aldrich).

### RNA isolation, cDNA synthesis and quantitative real-time PCR

Cells were lysed in TRIzol Reagent (Thermo Fisher Scientific, Massachusetts, USA), and 1/5 volume of chloroform was added for phase separation. Samples were centrifuged at 14,000 × *g* for 20 min and the aqueous phase was collected. RNA was precipitated by adding an equal volume of isopropanol to the aqueous phase followed by overnight incubation at -20 °C. The next day, samples were centrifuged 30 min at 14,000 × *g* at 4 °C. The supernatant was discarded, and samples were washed with 100% ethanol, followed by incubation with 0.1 M EDTA (Invitrogen, Massachusetts, USA) and 8 M Lithium Chloride (Merck, New Jersey, USA) overnight at -20 °C to remove hydroxyapatite and other minerals present in the extracellular matrix [5]. Then, samples were centrifuged for 30 min at 14,000 × *g* and 4 °C and washed three times with 70% ethanol, followed by a final wash in 100% ethanol. Finally, pellets were dissolved in RNase-free H_2_O. Total RNA concentration was determined using the NanoDrop 8000 Spectrophotometer (ThermoFisher Scientific, Massachusetts, USA). One µg of total RNA was reverse transcribed using the RevertAid First Strand cDNA Synthesis Kit (ThermoFisher Scientific) according to the manufacturer’s protocol. Gene expression was evaluated by quantitative real-time PCR using a QuantStudio 7 Flex Real-Time PCR system (Applied Biosystems, Massachusetts, USA) using SYBR green PCR master mix reagent (Promega, Wisconsin, USA). All primer sets were designed to span at least one exon-exon junction (Table 1). To calculate the relative expression of the genes of interest, the Ct values of the target genes were subtracted from the housekeeping gene acidic ribosomal phosphoprotein P0 (*36.b4)* to obtain the ΔCt. and expressed as 2^−ΔCt^.

**Table 1:** Primer sequences used for qPCR

Gene	Forward	Reverse
*36.b4*	TTGGCCAATAAGGTGCCAGC	GGAGGTCTTCTCGGGTCCTA
*Alpl*	ACACTCGGCCGATCGGGACT	CGCCACCCATGATCACGTCGA
*Bax*	GCTGATGGCAACTTCAACTG	GATCAGCTCGGGCACTTTAG
*Bcl2*	CGGAGGCTGGGATGCCTTTGT	AGTGATGCAGGCCCCGACCA
*Bglap*	CCTGAGTCTGACAAAGCCTTCAT	CAAGGTAGCGCCGGAGTCT
*Dmp1*	TGTGGGAAAAAGACCTTGGGAG	GTATCTGGCAACTGGGAGAGCA
*Fgf23*	CCATCAGACCATCTACAGTGCC	CTTCGAGTCATGGCTCCTGTT
*Hes1*	AAAATTCCTCCTCCCCGGT	ATGATAGGCTTTGATGACTTTCTG
*Hmox1*	GCCACCAAGGAGGTACACAT	AAGGAAGCCATCACCAGCTTA
*Nqo1*	GGTAGCGGCTCCATGTACTC	CGCAGGATGCCACTCTGAAT
*Postn*	GCTTCAGGGAGACACACCTGC	CTCTGTGGTCTGGCCTCTGGGT
*Runx2*	AAGTGCGGTGCAAACTTTCT	TCTCGGTGGCTGGTAGTGA
*Sost*	ACCTCCCCACCATCCCTATG	TGTCAGGAAGCGGGTGTAGTG

### Western blot

Cytoplasmic and nuclear protein was collected using NE-PER Nuclear and Cytoplasmic Extraction Reagents (Thermo Fisher) according to the manufacturer’s protocol. Equal amounts of protein were loaded and separated by SDS-PAGE (Bio-Rad Laboratories B.V., Veenendaal, The Netherlands) and transferred onto a polyvinylidine difluoride membrane (Amersham Hybond Western Blotting membranes, Sigma-Aldrich). Each membrane was blocked with 5% non-fat milk in Tris-buffered saline containing 0.1% Tween20 (TBS-T, Sigma-Aldrich) at room temperature for 1 h. The primary antibodies against FOXO3a (1:1,000, rabbit, PA5-27,145, Thermo-Fisher Scientific) and lamin B1 (1:1,000, rabbit, GTX103292, GeneTex, California, United States) were incubated overnight at 4 °C. After three washes with TBS-T the membranes were incubated with an anti-rabbit antibody (1:1,000, goat, 7074 s, Cell Signaling) conjugated with horseradish peroxidase (HRP) for 1 h at room temperature. The proteins were detected by an Amersham imager 600 (Amersham) using Western ECL Substrate (Bio-Rad Laboratories B.V.) and quantified using ImageJ (https://imagej.nih.gov).

### ROS assay

MC3T3-E1 cells were seeded in a 96-wells plate at density of 1,700 cells per well and differentiated as described above. At day 28, cells were stained with 2’,7’–dichlorofluorescein diacetate (DCFDA, Abcam) and treated with the indicated compounds. Afterwards ROS levels were measured using the DCFDA Cellular ROS Detection Assay Kit (ab113851, Cambridge, United Kingdom) according to the manufacturer’s protocol.

### Phosphate assay

Cells were collected in 20 mM Tris buffer and intracellular phosphate levels were measured using the Phosphate Assay Kit (Colorimetric) (ab65622, Abcam) according to the manufactures’ protocol.

### Statistics

The data are shown as mean ± standard error of mean (SEM) of representative experiments, in which n represents the number of individual samples within an experiment. All experiments were performed at least two times, with exception of the ROS assay. Data from secondary and tertiary experiments are reported in supplementary file 1. When individual datapoints could not be shown the data are shown as mean ± standard deviation. Two groups were compared using an unpaired student’s *t* test. More than two groups were tested for significance using a one-way analysis of variance (ANOVA) followed by the Tukey post-hoc test. When the effects of multiple factors were tested, a two-way ANOVA was used. Differences were considered significant if *p* < 0.05 and indicated as following: * *p* < 0.05, ** *p* < 0.01, *** < 0.001, **** *p* < 0.0001. All statistical analyses were done using R studio rstatix package (version 0.7.1).

## Results

### The ROS-response machinery is involved in the regulation of Fgf23

**Fig. 1:**
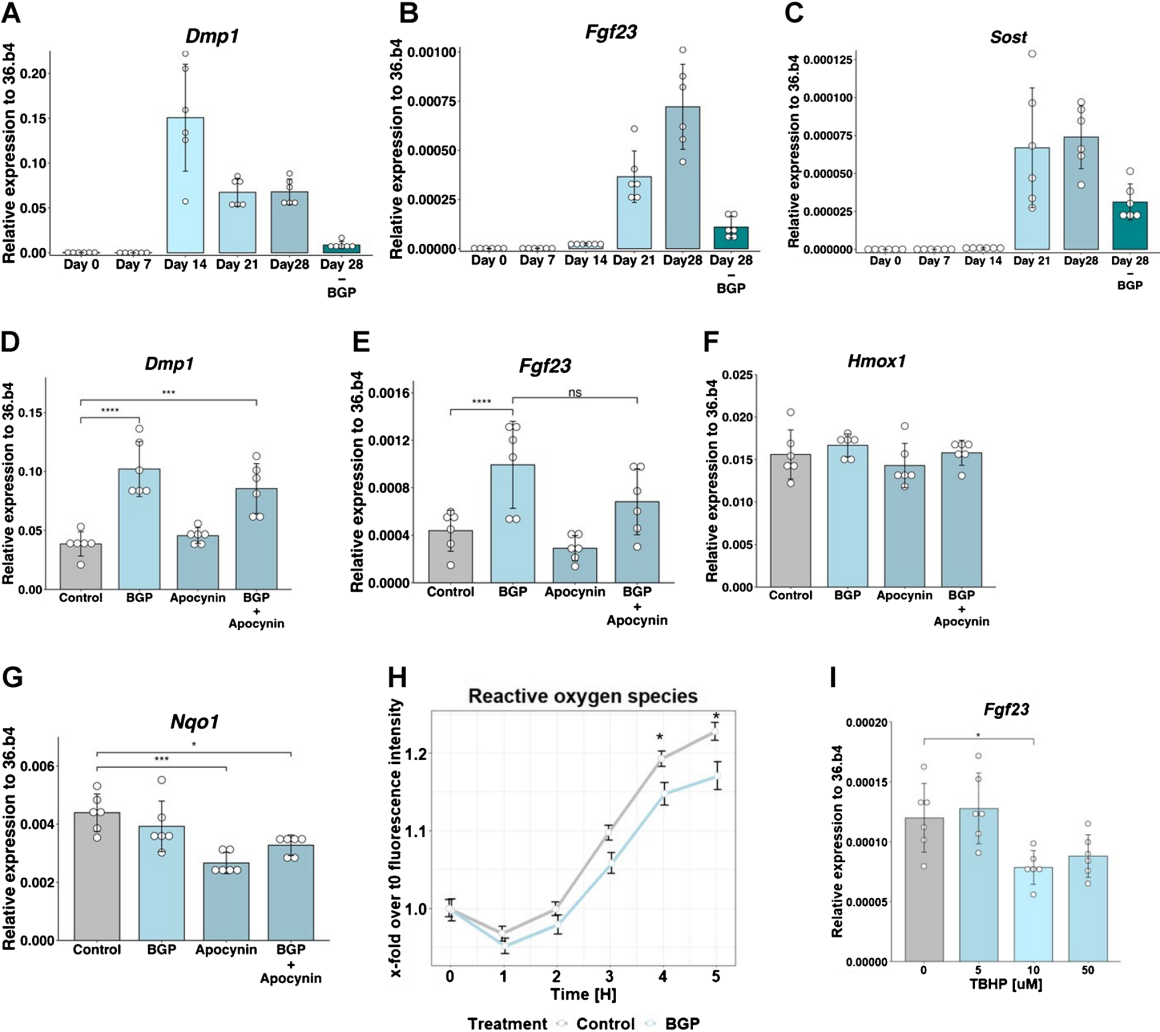
ROS-machinery is not involved in regulation of FGF23 by phosphate. Expression of (**A**) *Dmp1*, (**B**) *Fgf23* and (**C**) *Sost* in MC3T3-E1 cells at day 28 after 4 weeks of differentiation. Expression of (**D**) *Dmp1*, (**E**) *Fgf23*, (F) *Hmox1* or (**G**) *Nqo1* in MC3T3-E1 cells at day 28 after 24-h treatment with BGP, apocynin or a combination. (**H**) Formation of ROS after 0 – 5-h treatment with BGP. (**I**) Expression of *Fgf23* at day 28 with indicated concentrations of TBHP. Gene expression was normalized to expression of housekeeping gene *36.b4*. Error bars indicate mean ± SEM. Significance was indicated as follows: * *p* < 0.05, ** *p* < 0.01, **** *p* < 0.0001. (D-G: Two-way ANOVA followed by Tukey post-hoc test, H: unpaired student’s *t* test for every timepoint, I: one way ANOVA followed by Tukey post-hoc test.) Abbreviations: BGP: β-glycerophosphate, TBHP: tert-butyl hydroperoxide

MC3T3-E1 cells were differentiated into FGF23-expressing cells in four weeks, when they expressed early osteocyte marker dentin matrix protein 1 (*Dmp1*) and mature osteocyte markers, sclerostin (*Sost*) and *Fgf23* (Fig. 1A-C). At day 21, BGP was removed from the culture system to treat the cells for 24 h with BGP at day 27 without increasing the extracellular phosphate concentration above physiological levels, resulting in decreased expression of the phosphate responsive osteocyte markers. However, expression of the osteoblast markers *alkaline phosphatase* (*Alpl), Osteocalcin* (*Bglap*), *periostin* (*Postn*) and *runt-related transcription factor 2* (*Runx2*) were not affected by the withdrawal of BGP, indicating the cells are not dedifferentiating (Supplementary Fig. 1A-D). When these cells were treated with BPG, expression of phosphate response gene *Dmp1* was increased by 2.64-fold and *Fgf23* was increased by 2.27-fold (Fig. 1D and E). When apocynin, a NADPH oxidase inhibitor was used, thus preventing the formation of ROS, the expression of *Dmp1* was unaffected and the increase in *Fgf23* expression was lower (1.56-fold increase), although not significantly lower than BGP alone (Fig. 1D and E). Unexpectedly, the expression of both ROS-response genes *Nqo1*and *Hmox1* were unchanged after BGP treatment (Fig. 1F-G). However, we observed a significant interaction effect between phosphate and apocynin on *Nqo1* expression (F(1, 20) = 4.875, *p* = 0.0326). To study whether these genes were not regulated by BGP as BGP failed to stimulate ROS production, a ROS assay was performed. The results indicated a significant but modest decrease in ROS formation compared to the control (Fig. 1H). Finally, cells were treated with TBHP, a ROS inducing agent, to study whether ROS can directly stimulate *Fgf23* expression. None of the concentrations tested enhanced *Fgf23* expression, while 10 μM TBHP even significantly decreased the expression of *Fgf23* (Fig. 1I). Collectively, these results indicate that the ROS-regulatory machinery is not involved in the regulation of *Fgf23* following BGP treatment.

### Sirt1 is involved in the regulation of Fgf23 after BGP treatment

**Fig. 2:**
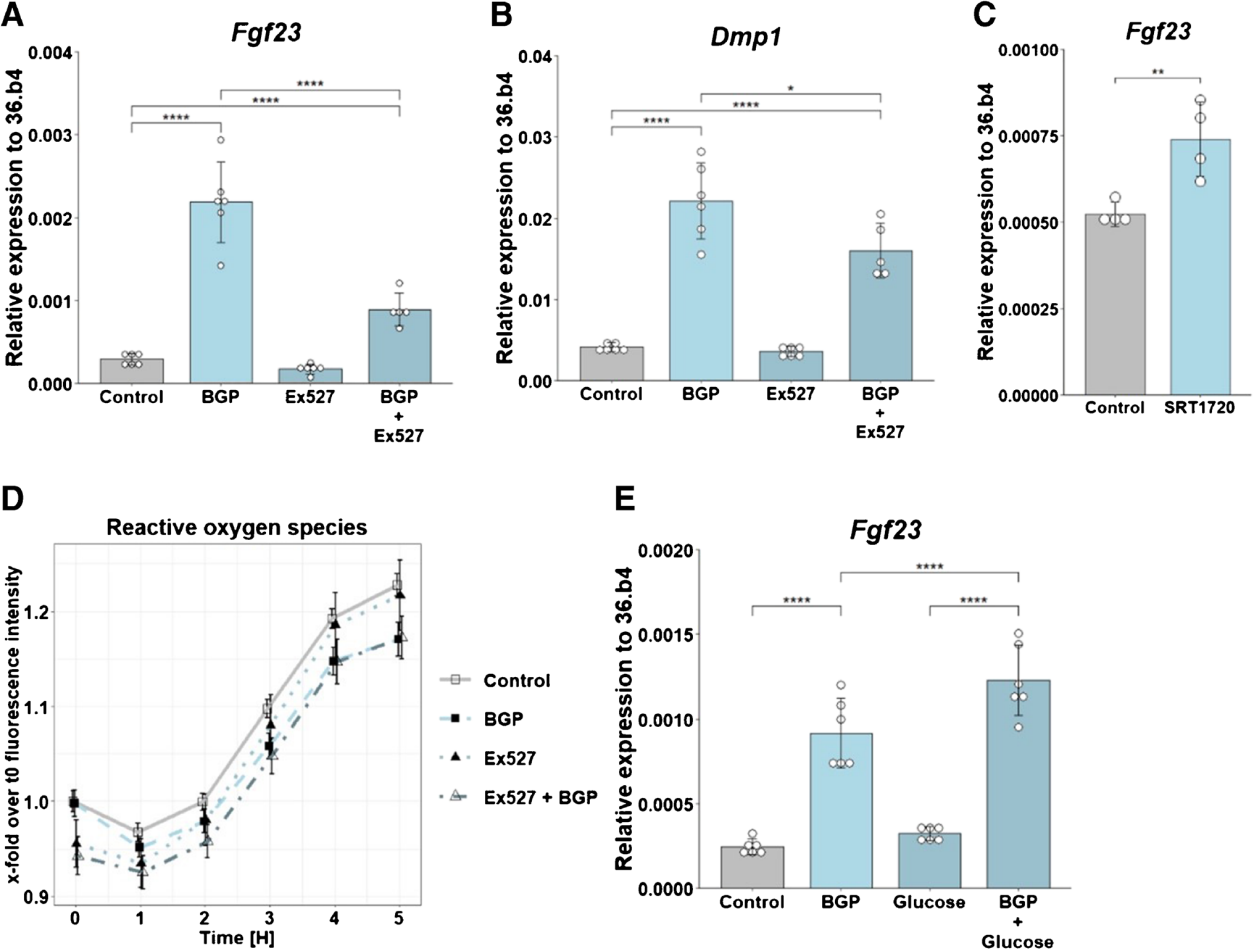
SIRT1 is a regulator of FGF23. Expression of (**A**) *Fg23* and (**B**) *Dmp1* MC3T3-E1 cells at day 28 after 24-h treatment with BGP, Ex527 or a combination. (**C**) Expression of Fgf23 at day 28 after 24-h treatment with SRT1720. (**D**) Formation of ROS after 0 – 5-h treatment with BGP, Ex527 or a combination, depicted as fold induction over t = 0 in the control situation, partly redrawn from Fig. 1H (n = 8). (**E**) Expression of *Fgf23* after treatment with BGP, glucose or a combination. Gene expression was normalized to expression of housekeeping gene *36.b4*. Two groups were compared using an unpaired student’s *t* test. Error bars indicate mean ± SEM. Significance was indicated as follows: * *p* < 0.05, ** *p* < 0.01, *** < 0.001, **** *p* < 0.0001. (A, B, E: Two-way ANOVA followed by Tukey post-hoc test, C: unpaired student’s *t* test, D: two-way ANOVA for every timepoint followed by Tukey post-hoc test.) Abbreviations: BGP: β-glycerophosphate

To test whether the nutrient sensor SIRT1 is involved in the regulation of FGF23 by BGP, SIRT1 inhibitor Ex527 was used in combination with BGP. Ex527 significantly attenuated the effect of BGP treatment on *Fgf23* expression. (Fig. 2A). The response of *Dmp1* to BGP was also decreased by Ex527 although this effect was less pronounced than the decrease in *Fgf23* expression (Fig. 2B). Moreover, a significant interaction effect from BGP and Ex527 was found on both *Fgf23* (F(1,20) = 27.24, *p* < 0.0001) and *Dmp1* (F(1,19) = 5.311, *p* = 0.0326). Activation of SIRT1 using SRT1720 resulted in a 1.4-fold increase of *Fgf23* (Fig. 2C). Ex527 did not affect the production of ROS in presence or absence of BGP, indicating that there is indeed no role for ROS in these processes (Fig. 2D). As SIRT1 is regarded as a glucose sensor and SIRT1 enhances *Fgf23*, we tested whether the addition of glucose changed the response of *Fgf23* to BGP. Increasing the glucose present in the culture medium by 10 mM resulted in slightly but significantly higher *Fgf23* expression in response to BGP, but there was no significant interaction between BGP and glucose (F(1, 20) = 3.61, *p* = 0.0719) (Fig. 2E), indicating that the effect of SIRT1 on *Fgf23* is independent of glucose levels. Overall, these findings highlight the regulatory role of SIRT1 in modulating *Fgf23* in response to BGP.

### Expression of Hes1 is increased after treatment with BGP

**Fig. 3:**
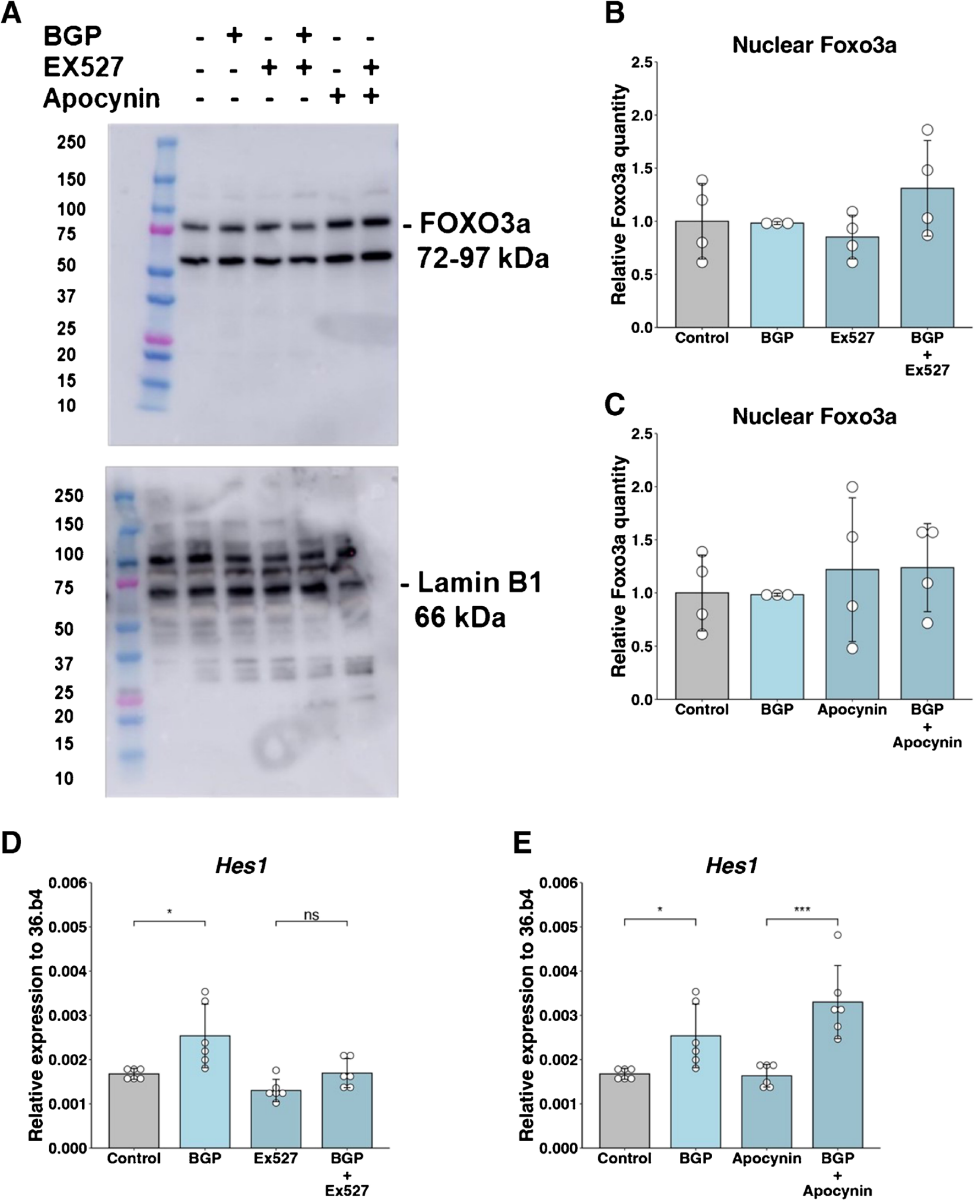
Effects of SIRT1 on FGF23 are not mediated by FOXO3a. (**A**) Representative western blot for FOXO3a and Lamin B1 MC3T3-E1 at day 28 after 24-h treatment with BGP, Ex527, apocynin or a combination. (**B-C**) Quantification of nuclear FOXO3a compared to Lamin B1. (**D-E**) Expression of *Hes1* in MC3T3-E1 cells at day 28 after 24-h treatment with BGP, Ex527, apocynin or a combination. Gene expression was normalized to expression of housekeeping gene *36.b4*. Error bars indicate mean ± SEM. Significance was indicated as follows: * *p* < 0.05, *** < 0.001. (B-E: Two-way ANOVA followed by Tukey post-hoc test.) Abbreviations: BGP: β-glycerophosphate

SIRT1 regulates FOXO3a by deacetylating it, enhancing its transcriptional activity and promoting its cellular functions involved in stress response and longevity [6]. To investigate the potential involvement of FOXO3a in the regulation of FGF23, nuclear translocation of FOXO3a was examined using Western blot analysis. BGP treatment did not affect nuclear FOXO3a levels, nor did treatment with Ex527 or apocynin affect the translocation of FOXO3a to the nucleus (Fig. 3A-C, Supplementary file 2A-B) This indicates that transcription of *Fgf23* after BGP treatment is not regulated by FOXO3a. Expression of transcription factor *Hes1,* a target of SIRT1 in neuronal cells, was increased by 1.51-fold after BGP treatment (Fig. 3D and E). When cells were treated with Ex527 and BGP, *Fgf23* was not significantly different expressed compared to treatment with Ex527 alone, indicating the involvement of SIRT1 in the increased *Hes1* expression. Inhibition of ROS using apocynin did not affect the expression of *Hes1*, indicating that ROS formation is not involved in this process (Fig. 3C).

### Phosphate transporters are essential for the expression of Fgf23

**Fig. 4:**
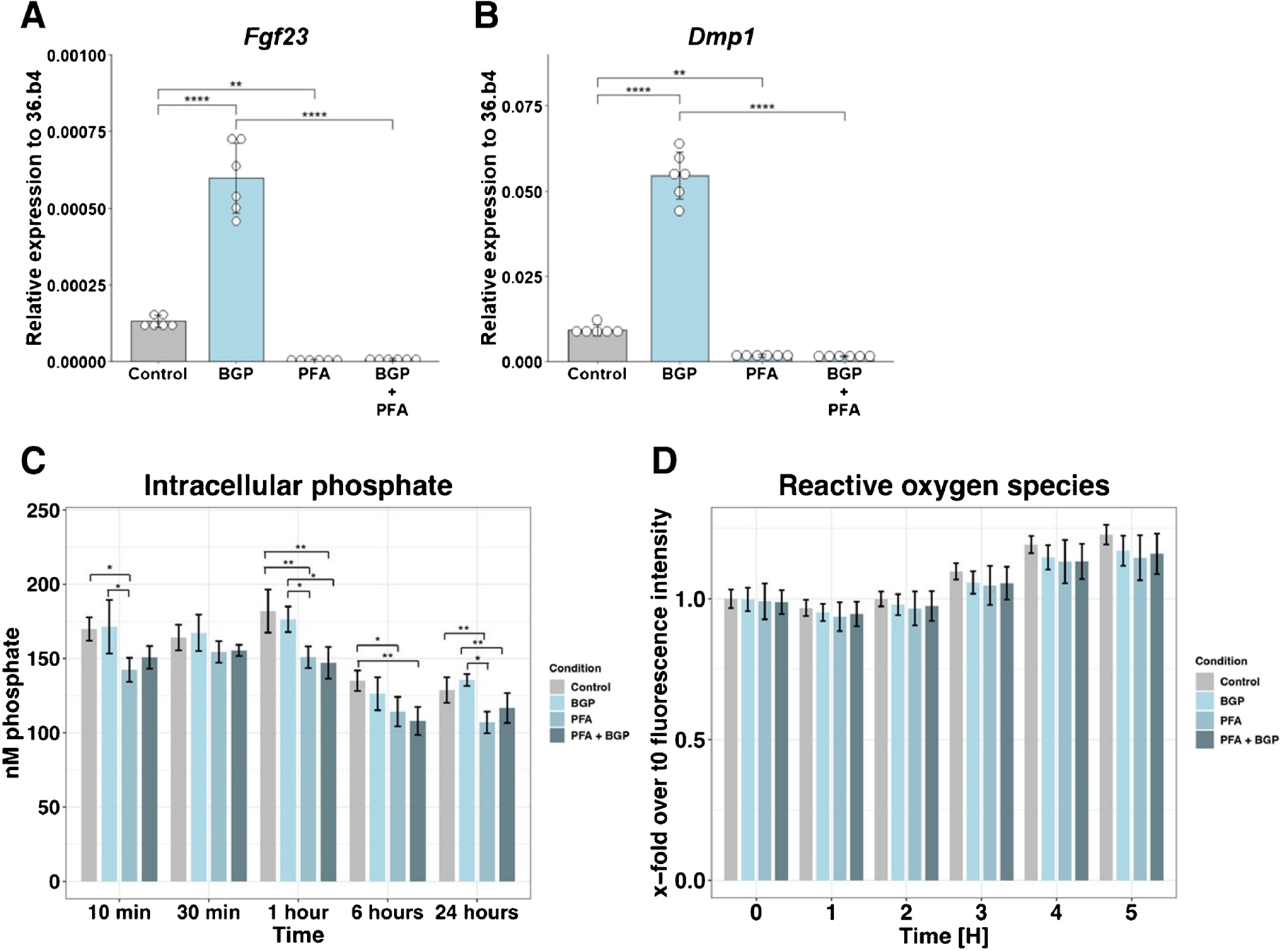
Phosphate transporters are essential for *Fgf23* transcription. Expression of (**A**) *Fg23*, (**B**) *Dmp1 1* in MC3T3-E1 cells at day 28 after 24-h treatment with BGP, PFA or a combination. (**C**) Intracellular phosphate levels in MC3T3-E1 cells after treatment with BGP, PFA or a combination for the indicated time points (n = 4). (**D**) Formation of ROS in MC3T3-E1 at day 28 after treatment with BGP, PFA or a combination for the indicated time points, partly redrawn from Fig. 1H (n = 8). Gene expression was normalized to expression of housekeeping gene *36.b4*. Error bars indicate mean ± SEM. Significance was in indicated as following: * p < 0.05, ** *p* < 0.01, *** p < 0.001, **** *p* < 0.0001. (A-B: Two-way ANOVA followed by Tukey post-hoc test, C-D: Two-way ANOVA followed by Tukey post-hoc test for every timepoint. Abbreviations: BGP: β-glycerophosphate, PFA: Sodium phosphonoformate tribasic hexahydrate

To study whether phosphate transporters are involved in FGF23 signaling, the general phosphate transporter inhibitor PFA was used. PFA completely abolished the expression of *Fgf23* and *Dmp1* both in the absence and presence of BGP (Fig. 4A-B). Expression of *Hmox1* and *Nqo1* was not affected by PFA, while the expression of the osteocyte marker *Sost* was decreased (Supplementary Fig. 2A-C). A significant interaction effect was observed between phosphate and PFA for the expression of *Fgf23* (F(1, 20) = 93.66, *p* < 0.0001), *Dmp1* (F(1, 20) = 239.3, *p* < 0.0001), and *Sost* (F(1, 20) = 5, *p* = 0.0369). The ratio of apoptosis markers *Bax* and *Bcl2* was unchanged by the PFA treatment, indicating that PFA does not affect apoptosis (Supplementary Fig. 2D). An intracellular phosphate assay was performed at 10 min, 30 min, 1 h, 6 h and 24 h after refreshing the cells with medium containing PFA and/or BGP. This revealed that cells with medium containing BGP did not lead to higher levels of intracellular phosphate compared to refreshing without BGP. However, treatment with PFA resulted in lower intracellular phosphate levels, both in the presence and absence of BGP (Fig. 4C). Additionally, PFA did not have a significant effect on ROS production by the cells (Fig. 4D). Together these data indicate that functioning phosphate transporters are required for the expression of phosphate-related genes in MC3T3-E1 cells.

## Discussion

We set out to explore a potential role for the ROS-SIRT1-HES1 axis in the regulation of *Fgf23* by BGP using MC3T3-E1 osteocyte-like cells. Our study revealed that regulation of *Fgf23* by BGP does not involve the ROS-response machinery. *Fgf23* expression increased with BGP treatment, which was not affected by inhibiting ROS formation through the NADPH oxidase inhibitor apocynin. Moreover, BGP did not induce ROS production, while direct induction of ROS by THBP also failed to increase *Fgf23* expression, indicating that ROS stimulation alone is insufficient to drive *Fgf23* expression. However, inhibition of SIRT1 hindered the response of *Fgf23* to BGP. Conversely, the activation of SIRT1 increased *Fgf23* expression, demonstrating its upstream role. Finally, BGP promoted the expression of *Hes1*, but not in the presence of Ex527, suggesting the potential involvement of HES1 in SIRT1-mediated *Fgf23* transcription following BGP treatment. However, the exact nature of the relationship between HES1 and FGF23, particularly in the context of regulation by BGP, remains unclear and warrants further investigation.

While our study confirmed that SIRT1 is involved in regulating *Fgf23*, it appears that this is not mediated through ROS as previously suggested. It's worth noting that earlier studies indicating direct regulation of *Fgf23* by ROS were conducted in UMR106 cells, an osteosarcoma cell line that may not reflect the physiology of *Fgf23* regulation in osteocytes. Additionally, these studies used inorganic sodium hydrogen phosphate in combination with 1,25(OH)_2_D_3_, whereas we used the organic phosphate BGP, which stimulated *Fgf23* expression in the absence of 1,25(OH)_2_D. Because MC3T3-E1 cells did not show increased ROS when treated with BGP, it is unlikely that organic phosphates increase ROS levels, but inorganic phosphates might increase ROS formation instead [9]. Despite BGP not increasing intracellular phosphate levels, our findings suggest that phosphate transporters are crucial for FGF23 production, as demonstrated by the complete absence of *Fgf23* transcription when cell were pre-treated with the general phosphate transporter inhibitor PFA. The decrease in ROS formation observed following treatment with the phosphate transporter blocker PFA [10] alone may be attributed to the reduced uptake of inorganic phosphate present in the cell culture media formulation.

Previously, SIRT1 has been associated with expression of *Fgf23* in response to glucose depletion, but to our knowledge it has not been associated with the response of *Fgf23* to BGP [24]. In a low glucose environment 5' AMP-activated protein kinase (AMPK) and SIRT1 activate the transcriptional co-activator peroxisome proliferator-activated receptor gamma coactivator 1-alpha (PCG1-α), which leads to transcription of the osteocytic genes *Fgf23*, *Dmp1* and *Sost*. Transcription factor FOXO3a has been implicated in the regulation of phosphate homeostasis and the expression of other genes involved in mineral metabolism, which could potentially overlap with the FGF23 signaling pathway [33]. However, we did not find any changes in the translocation of FOXO3a in response to BGP, suggesting that it is not involved in the regulation of *Fgf23*. It is possible that *Fgf23* is regulated by the transcription factor PCG-α in response to phosphate, similar to how it is regulated by glucose depletion [24]. Another potentially involved transcription factor is *Hes1* as it was upregulated by BGP, an event that was prevented by Ex527. There is currently no direct evidence of *Hes1* binding to *Fgf23*, but previous associations found between *Hes1* and *Fgf23* make it an interesting gene to study further in the context of *Fgf23* regulation by phosphate [29].

While previous studies have suggested a role for SIRT1 in regulating *Fgf23* in osteocytes, our study is the first to demonstrate that blocking SIRT1 activity prevents the regulation of *Fgf23* by phosphate, indicating a direct involvement of SIRT1 in the response of *Fgf23* to phosphate [24]. Interestingly, we observed that the effect of Ex527 on regulation of *Dmp1* by BGP was less pronounced than the effect on *Fgf23*, suggesting a mechanism that is at least partially specific for *Fgf23* regulation [15]. We have not fully elucidated the precise mechanism by which SIRT1 regulates *Fgf23* since the upstream factors leading to SIRT1 activation and involvement of transcription factors remain unknown. Moreover, as our experiments were carried-out in osteocyte-like MC3T3-E1 cells, further investigations using diverse in vitro and in vivo models are required to validate and expand upon our observations.

Even though our study does not fully describe how SIRT1 regulates *Fg23* after BGP treatment, it did yield new insights in the regulation of *Fgf23* by phosphate. Additionally, it highlights several intriguing opportunities for future research. Although *Hes1* shows a similar regulation to *Fgf23*, direct evidence of its involvement in *Fgf23* regulation is lacking, rendering this an interesting subject for further research. Moreover, the role of phosphate transporters as signaling receptors would be an interesting direction for future research. We have shown that blockage of the phosphate transporters results in diminished expression of *Fgf23*, both in the presence and absence of phosphate. Interestingly, the administration of BGP did not increase intracellular phosphate levels. BGP is rapidly degraded to inorganic phosphate when it is added to cell culture medium, which easily can be taken up by the cells [2]. It is therefore remarkable that BGP did not increase intracellular phosphate levels. This observation raises an important notion whether BGP binding to transporters at the cell surface, preventing uptake, may serve as a signaling mechanism for downstream pathways, leading to FGF23 regulation [4].

In conclusion, our study demonstrates the involvement of the ROS-SIRT1-HES1 axis in regulating *Fgf23* in response to phosphate in MC3T3-E1 cells. However, contrary to previous results, we found that ROS do not mediate this regulation. These findings highlight the complex nature of FGF23 regulation by (organic) phosphates and the need for further investigations to unravel the upstream mechanism and transcription factors responsible for SIRT1-mediated regulation of *Fgf23*. Overall, our study contributes to the current understanding of the extra- and intracellular mechanisms regulating the response to phosphate in osteocytes. Future studies in animals with altered phosphate levels should prove if the Sirt1 pathway is indeed a potential target when dealing with disturbed phosphate homeostasis. Understanding these molecular pathways could pave the way for interventions aimed at modulating *Fgf23* levels, offering potential clinical applications in the management of disorders associated with chronically high phosphate levels.

### Supplementary Information

Below is the link to the electronic supplementary material.

Supplementary file1 Expression of osteoblast markers during MC3T3-E1 differentiation. Expression of (A) Alpl, (B) Bglap, (C) Postn and (D) Runx2  in MC3T3-E1 during differentiation. In week 4 cells were differentiated with and without BGP. Error bars indicate mean ± SEM. Abbreviations: BGP: β-glycerophosphate. (XLSX 27 KB)

Supplementary file2 Effect of PFA on gene expression. Expression of (A) Hmox1, (B) Nqo1 and (C) Sost in MC3T3-E1 cells at day 28 after 24-hour treatment with BGP, PFA or a combination. (D) Ratio for relative expression of Bax and Bcl2 in MC3T3-E1 cells at day 28 after 24-hour treatment with BGP, PFA or a combination. Error bars indicate mean ± SEM. Significance was in indicated as following: ** p < 0.01, *** p < 0.001, **** p < 0.0001. (A-D: Two-way ANOVA followed by Tukey post-hoc test) Abbreviations: BGP: β-glycerophosphate, PFA: Sodium phosphonoformate tribasic hexahydrate. (PDF 423 KB)

## Data Availability

Not applicable.
