# Supplementary material for: A role for sirtuin 1 in FGF23 activation following β-glycerophosphate treatment
Source: Pflugers Arch. 2024 May 21;476(8):1279–88. doi: 10.1007/s00424-024-02974-5 (PMC11271368; doi:10.1007/s00424-024-02974-5)
Supplement: Supplementary file 2 — Supplementary file2 Effect of PFA on gene expression. Expression of (A) Hmox1, (B) Nqo1 and (C) Sost in MC3T3-E1 cells at day 28 after 24-hour treatment with BGP, PFA or a combination. (D) Ratio for relative expression of Bax and Bcl2 in MC3T3-E1 cells at day 28 after 24-hour treatment with BGP, PFA or a combination. Error bars indicate mean ± SEM. Significance was in indicated as following: ** p < 0.01, *** p < 0.001, **** p < 0.0001. (A-D: Two-way ANOVA followed by Tukey post-hoc test) Abbreviations: BGP: β-glycerophosphate, PFA: Sodium phosphonoformate tribasic hexahydrate. (PDF 423 KB) [file 424_2024_2974_MOESM2_ESM.pdf]

A

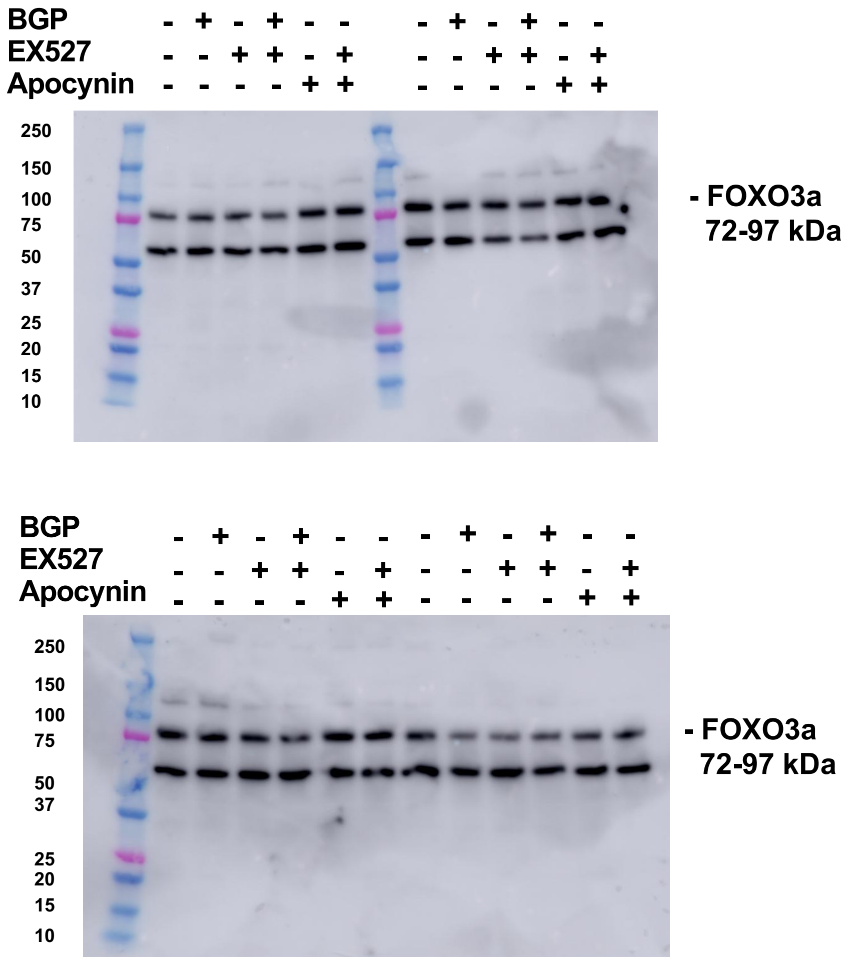

BGP
EX527
Apocynin
250
150
100
75
50
37
25
20
15
10
- FOXO3a
72-97 kDa

B

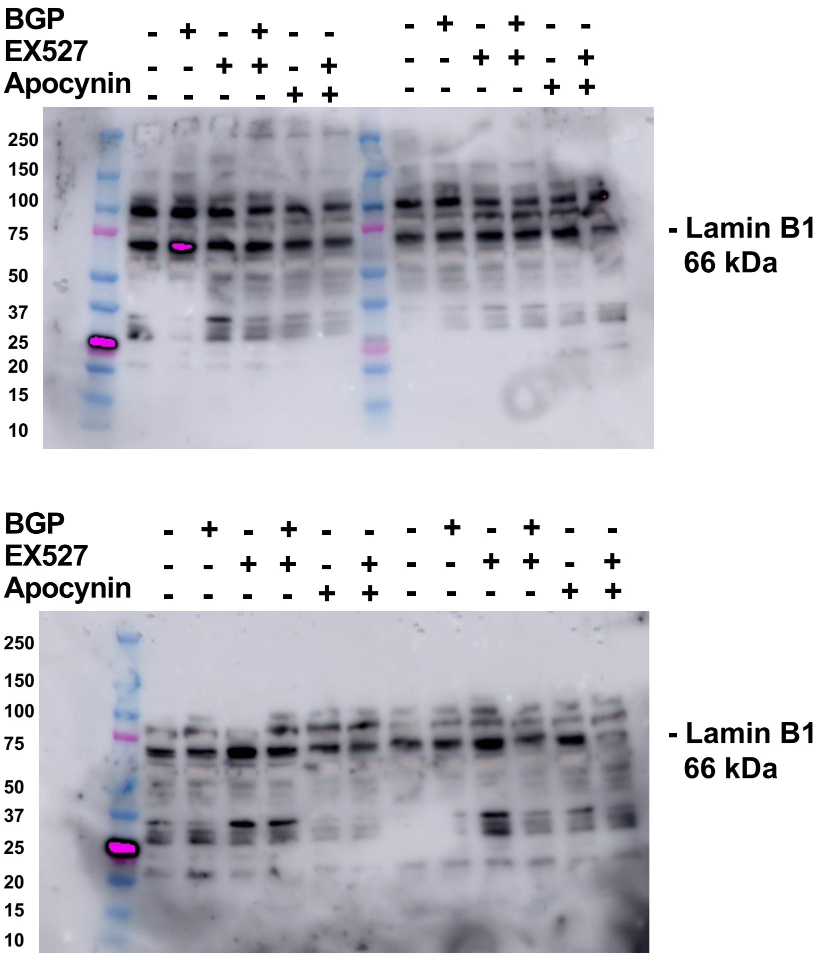

BGP
EX527
Apocynin
250
150
100
75
50
37
25
20
15
10
- Lamin B1
66 kDa
